# Analysis of histological features and recurrence risk assessment of papillary thyroid carcinoma according to presurgery FNAC category

**DOI:** 10.1007/s13304-025-02121-4

**Published:** 2025-02-26

**Authors:** Sium Wolde Sellasie, Stefano Amendola, Leo Guidobaldi, Tommaso Piticchio, Isabella Nardone, Simona Zaccaria, Giovanni Tacchi, Francesco Pedicini, Luigi Uccioli, Pierpaolo Trimboli

**Affiliations:** 1 Division of Endocrinology and Diabetes, Department of Biomedicine and Prevention, CTO Andrea Alesini Hospital, University of Rome Tor Vergata, 00133 Rome, Italy; 2 PhD School of Applied Medical-Surgical Sciences, University of Rome Tor Vergata, 00133 Rome, Italy; 3 UOC of Pathologic Anatomy and Cytodiagnostic, Sandro Pertini Hospital, ASL RM2, 00157 Rome, Italy; 4 Endocrinology Section, Department of Clinical and Experimental Medicine, Garibaldi Nesima Hospital, University of Catania, Catania, Italy; 5 Department of Medicine and Surgery, University Kore of Enna, Enna, Italy; 6 Thyroid Endocrine Surgery, Sant’Eugenio Hospital, 00144 Rome, Italy; 7 Servizio di Endocrinologia e Diabetologia, Ente Ospedaliero Cantonale (EOC), Lugano, Switzerland; 8 Facoltà di Scienze Biomediche, Università della Svizzera Italiana (USI), Lugano, Switzerland

**Keywords:** Papillary thyroid carcinoma, ATA risk score, Fine needle aspiration, Cytology

## Abstract

Identifying preoperatively cases of more indolent papillary thyroid carcinoma (PTC)could be of high interest. The aim of this study was to verify previously published data on the prognostic value of fine needle aspiration cytology (FNAC)in PTC, also comparing findings from high-volume (HV)and low-volume (LV)institutions. From January 2022 to June 2024, the institutional database of the endocrinological surgery unit of Sant’Eugenio Hospital (Rome, Italy)was retrospectively reviewed to select patients who underwent thyroid surgery for PTC. To evaluate the prognostic value of presurgical FNAC, all histological features and the ATA risk of the study groups were compared. Later, data of patients entirely managed at our institution, considered as an HV institute, were compared with that of cases operated at our institution following FNAC performed LV centres.The 159 PTC nodules included were classified as TIR3B (20.1%),TIR4 (32.7%),and TIR5 (47.2%).The distribution of FNAC report between HV and LV was different (*p* = 0.01). The presence of lymph node metastasis (*p* = 0.004), and peri-thyroid tissue invasion (*p* = 0.02)increased according to the FNAC category. Significant difference among the three FNAC categories was also observed in PTC subtype (*p* = 0.006)and Hashimoto’s thyroiditis (*p* = 0.02).In addition, a significant different trend was found in ATA risk assessment, being the risk of recurrence more prevalent according to the FNAC category (*p* = 0.008). According to the second study aim, the higher prevalence of low-risk cases in TIR3B was confirmed in both HV (*p* = 0.04) and LV (*p* = 0.03)subgroups.PTCs with preoperative TIR3B have different histological features and ATA risk assessment with respect to cases with presurgical FNAC of TIR4/5.Particularly, PTC from TIR3B should have a pattern of more indolent cancers. As non-negligible extension, this data is not influenced by the institutional setting with high or low thyroid-FNAC volume.

## Introduction

The prevalence of thyroid nodules (TN) in adults ranges from 16 to 68%, depending on the study and population examined [1]. In recent years, the widespread use of ultrasound (US) and other radiological techniques [2] has allowed the detection of non-palpable TN that may require further diagnostic process, mainly fine needle aspiration cytology (FNAC). The latter holds a crucial role in TN management because of its high reliability in distinguishing malignant from benign TN [3], or at least improving the estimation of the risk of malignancy (ROM).

Fortunately, approximately 90% of TN are benign and 95% of TN patients are asymptomatic on diagnosis and over follow up [4]. In the last decades, it was observed an increased diagnosis of TNs, and malignant cases as a consequence [5], which has been responsible for surgical treatments increase. This trend has enabled the acquisition of important knowledge about thyroid tumours, optimising their managements, as confirmed by the stability of thyroid-related mortality rate [6].For these reasons, main international guidelines [7, 8] are in favour of a careful diagnostic work-up with rule-out strategy, and recommend a “less is more” therapeutic approach with the aim of reducing overdiagnosis, overtreatment, and both related implications and potential complications. With these premises, thyroidologists are asked to care (I) a tailored surgery, reducing prophylactic total thyroidectomy and central cervical lymph nodes dissection, and, ideally, (II) implement non-surgery management options such as mini-invasive treatment or active surveillance.

Identifying preoperatively cases of more indolent papillary thyroid carcinoma (PTC) could be of high interest [9]. However, defining the histological type of malignancy on FNAC is generally non-feasible, and understanding cancer subtypes is usually not possible and, in any case, unreliable [10]. In this view, the diagnosis and the management of the indeterminate TN (ITN) on FNAC is particularly critical for several reasons. First, discriminating malignant TN requiring treatment from benign lesions to be addressed to follow-up is possible only at histological evaluation after surgery. Second, non-negligible inter-observer variability between pathologists was found, as shown by a previous important study [11]. Third, there is a significant difference in assessing FNAC samples between low- (LV) and high-volume (HV) institutions, the latter tending to provide more likely a definitive interpretation [12]. Although this caveat, efforts have been made and the results should indicate that ITN at low risk of malignancy harbour carcinomas less aggressive than those with preoperative FNAC reports of suspicious of or consistent with malignancy (i.e., categories V-VI or TIR4-TIR5). These data prompt us to further investigate the matter by new studies aimed to evaluate whether we attempt (1) stage thyroid carcinomas before surgery and then 2) tailor as much as possible their therapy.

Taking into account these findings, the aim of this study was to verify previously published data on the prognostic value of cytology in papillary thyroid cancers (PTC), also comparing findings from HV and LV institutions.

## Materials and methods

### Institutional setting and management of TNs

The endocrinological surgery unit of Sant’Eugenio Hospital (Rome, Italy) can be considered as HV institute for thyroid disease, representing the surgery spot of a large area of community care for thyroid disease in Rome. Patients who refer to this institution for thyroid cancer or goiter surgery have already undergone general thyroid hormonal assessment (i.e., TSH, plus FT3 and FT4 when indicated, and Calcitonin), US evaluation with TN assessment according to ACR TI-RADS score system [13], and usually FNAC with cytological smears classified according to ICCRTC [14]. The latter includes seven categories: inadequate (TIR1), inadequate cystic (TIR1C), not neoplastic (TIR2), low-risk (TIR3A), and high-risk (TIR3B) indeterminate, suspicious of (TIR4) and consistent with (TIR5) malignancy. Specifically, the overall resection rate is significant in patients with TIR3B, TIR4 or TIR5 while only a minor part of cases with TIR1 to TIR3A is operated [15].

### Case Selection

The study period was January 2022-June 2024. The institutional database was retrospectively reviewed to select patients who underwent thyroid surgery. The inclusion criteria were: (I) age > 18 years; (II) availability of pre-surgery data, including clinical, US and FNAC; (III) FNAC report of TIR3B, TIR4 or TIR5; (IV) histological outcome of PTC. Incidental PTCs not corresponding to the biopsied TN were excluded. All patients signed the informed consent and privacy forms.

### Measure and reference standard

The included patients were divided into three major groups such as TIR3B, TIR4, and TIR5. To evaluate the prognostic value of the presurgical FNAC report, all histological features (major diameter of cancer, multifocality and number of foci, bilaterality, presence and number of lymph nodes metastases [LNM], thyroid capsule or peri-thyroid tissue invasion, vascular embolization, Hashimoto’s thyroiditis [HT], PTC subtype) and the category of recurrence risk according to ATA [7] of the study groups were compared. Later, data of patients entirely managed at our institution (from initial assessment to surgery) were compared with that of cases operated at our institution following FNAC-performed LV centres (i.e., < 300 FNACs per year), where it could be hypothesized downgraded cytological diagnosis, with higher ITN prevalence in favour of more definitive interpretation, such as TIR4/TIR5.

#### Statistical analysis

Continuous variables, such as age, major diameter of the primary PTC focus, number of PTC foci and the number of LNM were expressed as median and interquartile ranges (IQR) and compared by the Mann–Whitney *U* test. ACR TI-RADS classification, sex, multifocality, bilaterality, LNM presence, surgery intervention, thyroid capsule and/or peri-thyroid tissue invasion, vascular embolization, HT, histological subtype and ATA risk were expressed as frequencies and compared using the *x*^2^-test.

A *p* value of < 0.05 was considered to define statistical significance. All statistical analyses were performed with Jamovi software version 2.3 retrieved from https:// www.jamovi.org.

## Results

A total of 159 PTCs were included in this study. As detailed in Table 1, the sample was predominantly composed of women (78.7%), and the median age of the population was 50.5 years. The included TN were mainly assessed as ACR TI-RADS 4 (52.7%) or 5 (43.8%) and had a median maximum diameter of 9 mm. The distribution of the cytological report according to ICCRTC showed TIR3B in 20.1%, TIR4 in 32.7%, and TIR5 in 47.2%. Most FNAC samples (62.3%) were read by HV. When comparing these general characteristics between the HV and LV series, the distribution of the FNAC report was statistically significantly different (*p* = 0.01). Instead, no statistically significant difference was found in sex (*p* = 0.74), age (*p* = 0.05), ACR TI-RADS (*p* = 0.79), and lesion size (*p* = 0.74).

**Table 1:** Descriptive pre-surgery features, and comparative analysis between HV vs LV pathological units

	Total	HV pathologic unit (*n* = 99)	LV pathologic unit (*n* = 60)	*p*
Sex				0.74
Male	21.3%	22.2%	20%	
Female	78.7%	77.8%	80%	
Age (Median ± IQR)	50.5 (41–61)	53 (41–63)	48 (40–58.5)	0.05
ACR TI-RADS				0.79
3	3.5%	3.2%	5.9%	
4	52.7%	53.6%	47%	
5	43.8%	43.2%	47.1%	
FNAC reports				0.01
TIR3B	20.1%	13.1%	31.7%	
TIR4	32.7%	33.3%	31.6%	
TIR5	47.2%	53.6%	36.7%	
Lesion size (mm) (Median ± IQR)	9 (6.5–12)	9 (7–11.8)	9 (6–14)	0.74

Table 2 resumes histological features and ATA risk assessment according to the FNAC report. Hemithyroidectomy was more prevalent among TIR3Bs than the other classes (*p* < 0.01). PTC nodule’s size (*p* = 0.02), presence of LNM (*p* = 0.004), peri-thyroid tissue invasion (*p* = 0.02) increased according to the FNAC category. Significant difference among the three FNAC categories was also observed in PTC subtype (*p* = 0.006) and HT (*p* = 0.02). In addition, a significantly different trend was found in ATA risk assessment, being the risk of recurrence more prevalent according to the FNAC category (*p* = 0.008).

**Table 2:** Descriptive and comparative analysis of histological features according to cytology classes TIR3B, TIR4, and TIR5

	TIR3B	TIR4	TIR5	*p*
Surgery				< 0.01
Lobo-isthmectomy	37.5%	1.9%	4%	
Total thyroidectomy	62.5%	98.1%	96%	
PTC size (Median ± IQR)	6.5 (3–11.5)	9 (6.5–11)	10 (7–14)	0.02
Multifocality				0.05
Yes	31.3%	55.8%	54.7%	
No	68.7%	44.2%	45.3%	
Bilaterality				0.29
Yes	80%	66.7%	82.9%	
No	20%	33.3%	17.1%	
Number of foci (Median ± IQR)	3 (2–4)	3 (2–4)	2 (2–3)	0.13
LNM				0.004
Yes	3.1%	25%	33.3%	
No	96.9%	75%	66.7%	
Number of LNM (Median ± IQR)	1 (1–1)	3 (2–4)	4 (1–9)	0.55
Thyroid capsule invasion				0.09
Yes	43.7%	46.2%	62.7%	
No	56.3%	53.8%	37.3%	
Peri-thyroid tissue invasion				0.02
Yes	25%	30.8%	50.7%	
No	75%	69.2%	49.3%	
Vascular embolization				0.25
Yes	9.4%	11.5%	20%	
No	90.6%	88.5%	80%	
HT				0.02
Yes	37.5%	69.2%	56%	
No	62.5%	30.8%	44%	
PTC subtype				0.006
Follicular	78.1%	46.2%	29.3%	
Classic	18.8%	50%	61.3%	
Tall cell	0%	0%	4%	
Solid trabecular	3.1%	1.9%	2.7%	
Diffuse sclerosing	0%	1.9%	2.7%	
ATA risk				0.008
Low	81.3%	51.9%	45.3%	
Medium	18.7%	46.2%	48%	
High	0%	1.9%	6.7%	

*LNM* lymph-nodes metastasis, *PTC* papillary thyroid cancer, *HT* hashimoto's thyroiditis

**Fig. 1:**
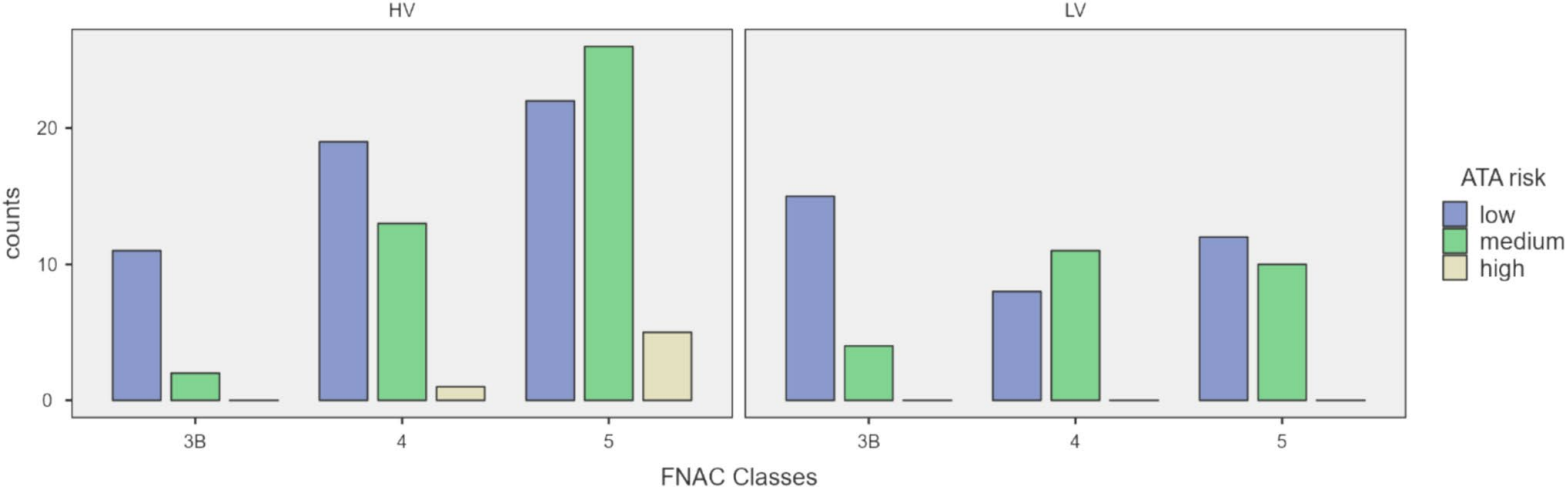
ATA risk and FNAC classes distribution according to HV and LV pathological unit

Figure 1 illustrates the analysis of the data according to the second study aim. The higher prevalence of low-risk cases in TIR3B was confirmed in both HV (*p* = 0.04) and LV (*p* = 0.03) subgroups. In the HV series, the ATA risk assessment was significantly different only between TIR3B and TIR5 (*p* = 0.02). In the LV series, the ATA risk assessment was significantly different only between TIR3B and TIR4 (*p* = 0.02).

## Discussion

Recent insights from the literature show a more indolent behaviour of ITN-PTC than TIR 4/5-PTC [16]. This should mean that we might start managing DTC patients before surgery according to their FNAC report. However, a major limit of thyroid cytology is the suboptimal inter-observer agreement between pathologists [11]. From this point of view, the institutional work volume can hold a role. Aim of this study was to analyse the FNAC report as a potential prognostic factor and then evaluate the role of the institutional setting in this context. The herein observed results can be summarized as follows. First, cancer size, peri-thyroid and neck invasion, and prevalence of classic and aggressive subtypes increase progressively with the highest FNAC categories. Second, even if the baseline characteristics of TN between HV and LV institutions are quite similar with the exception of FNAC category prevalence, minor differences were observed when compared the data from the two subgroups regarding the ATA risk assessment.

The study results merit a clinically oriented discussion. The present data corroborate and extend the results recently observed by other studies [16, 17]. The PTCs with preoperative TIR3B FNAC had less aggressive features than those with TIR4/TIR5 FNAC. In particular, the latter subgroup had a higher rate of neck LNM and peri-thyroid invasion than the former. In addition, in line with the paper by Croce et al. [16], the PTCs with preoperative TIR4/TIR5 FNAC had a worse assessment according to ATA risk stratification for cancer relapse. This data was novel information highlighted by Croce et al. [16] and we can confirm it using the same classification system for thyroid FNAC. As an extension of the previously published findings, the present found similar results even if only one category of ITN was included. In fact, Endo et al. [17] included Bethesda III and IV while Croce et al. [16] TIR3A and TIR3B. As a non-negligible difference between Bethesda system [10] and Italian consensus [14], the category TIR3B of the latter includes FNAC samples with nuclear atypia suggestive for PTC that in the Bethesda system are included in category III. On one hand, this data corroborates the conceptualization of the Italian consensus [14]. On the other hand, this data could somewhat explain the similar results found by Endo et al. [17] and Croce et al. [16]; the Bethesda category III and the category TIR3B could be the major influencers of their results, respectively. Basically, when looking at ITNs as a unique category, the two systems are comparable while their “subcategories” of ITN can significantly differ in terms of cancer risk [15]. As previously hypothesized [18], the progressive indolent behaviour, moving from TIR5-PTC to TIR3B-PTC, could be explained by the limited number of dedifferentiation steps undergone, in fact, on FNAC specimen, in TIR4/5 progressive cytological features of tumour progression, such as nuclear atypia, high cellularity with scant colloid occurred more. Therefore, it is reasonable to think that thyroid cancer progression and aggressiveness require the progressive accumulation of molecular alterations involving genes responsible for cell proliferation, differentiation, and apoptosis over time; FNAC specimen could reflect these alterations, enabling the prediction of the prognosis of PTC. In addition to this, another indirect sign of cell proliferation and growth such as PTC size, which is smaller in the TIR3B group, supports the different histological presentation between the FNAC classes. In this scenario, the impact of nodule’s size and coexistence of HT, significantly different between ITN and TIR4/TIR5, once again seems to impact positively on the cancer progression [19]. That PTC with preoperative TIR3B should have more indolent behaviour is further supported by the higher frequency of less aggressive subtypes in that FNAC category than the other ones (TUR4/TIR5). Remarkably, this finding was observed also in previous studies [20].

Finally, the result of the comparison between HV and LV institutions should be addressed. Importantly, the distribution of the FNAC report was significantly different, with TIR3B higher in LV and TIR5 in HV centers. These data could reflect the differences between the expertise of cytopathologists in detecting PTC and between the expertise of the aspirators in collecting an adequate and suitable sample. It is important to emphasise that the majority of PTCs were classified as moderately/highly suspicious, as demonstrated by a previous study [21] conducted in our center, which confirmed the performance of ACR TI-RADS.

The limits of this study could be resumed with the retrospective and monocenter study design, responsible for the sample size and potential bias, such as the more conservative surgical approach of TIR3B-PTCs, in which less lymph node dissection and total thyroidectomy were performed. To achieve a complete evaluation of whether PTC behaviour could be predicted by cytology, longitudinal and larger studies will be needed.

## Conclusions

This study confirms and extends the important insights from other previously studies; PTCs with preoperative ITN have different histological features and ATA recurrence risk assessment with respect to cases with presurgical FNAC of suspicious for or consistent with malignancy. Particularly, PTC from ITN should have a pattern of more indolent cancers. As non-negligible extension, this data is not influenced by the institutional setting with high or low thyroid-FNAC volume. The pre-treatment decision-making should consider these findings. These present and previous insights should be validated in larger cohorts.

## Data Availability

Database generated during and/or analyzed during the current study is available from the corresponding author upon reasonable request.
